# Trends of HIV indicators in Egypt from 1990 to 2021: time-series analysis and forecast toward UNAIDS 90–90–90 targets

**DOI:** 10.1186/s12889-023-15490-5

**Published:** 2023-04-01

**Authors:** Ramy Mohamed Ghazy, Salah Al Awaidy, Sarah Hamed N. Taha

**Affiliations:** 1 Tropical Health Department, High Institute of Public Health, Alexandria University, Alexandria, Egypt; 2 Health Affairs, Ministry of Health, Muscat, Oman; 3 Forensic Medicine and Clinical Toxicology Department, Faculty of Medicine, Cairo University, Cairo, Egypt

**Keywords:** HIV, Egypt, ART, 90–90–90 targets, Timeseries and forecasting, PLHIV

## Abstract

**Background:**

Infection with Human immunodeficiency virus (HIV) and the development of acquired immunodeficiency syndrome (AIDS) pose severe threats to public health across the world. This study aimed to describe and forecast the trend of HIV indicators, including progress towards the 90–90–90 targets in Egypt since 1990.

**Methods:**

The HIV indicators were graphically described, where the X axis is the time in a year and the Y axis is the value of the selected indicator for each year using data retrieved from UNAIDS. We used the Autoregressive Integrated Moving Average (ARIMA) model to forecast different HIV indicators from 2022 to 2024.

**Results:**

Since 1990, HIV prevalence has been < 0.01, the number of people living with HIV (PLHIV) has increased from < 500 to 30,000 with a higher male predominance since 2010, and the number of children living with HIV has increased from < 100 to 1100. The number of pregnant women who needed antiretroviral treatment (ART) to prevent maternofetal HIV transmission increased from < 500 during 2010–2014 to 780 in 2021, the percentage of women who received ART increased from 3% in 2010 to 18% in 2021, the number of children exposed to HIV who did not get infection increased from < 100 in 1990–1991 to 4900 in 2021. The number of AIDS-related deaths increased from < 100 in 1990 to < 1000 in 2021. Based on forecasting, we expect that by 2024 the number of PLHIV will be 39,325(95%CI, 33,236–37,334), 22% (95%CI, 13.0%–32.0%) of pregnant females will have access to ART, 6100(95%CI, 5714–6485) HIV exposed children will not be infected, 77.0%(95% CI 66.0%–86.0%) of the population who knew their HIV status, and 71.0% (95%CI, 61.0%–81.0%) among those who know their HIV status will be on ART.

**Conclusion:**

HIV is moving forward fast, however, the Egyptian health authority implements different control measures to control its spread.

## Introduction

Human immunodeficiency virus (HIV) infection and the development of acquired immunodeficiency syndrome (AIDS) pose severe threats to public health across the world. At the end of year 2021, the Joint United Nations Program on HIV/AIDS (UNAIDS) estimated that the worldwide prevalence of HIV among adults was 0.7% with 650,000 AIDS-related deaths [1]. Sub-Saharan Africa (SSA) is the region that continues to be the most severely impacted by HIV, with almost 1 in 25 adults living with the virus. SSA accounts for two-thirds of all people living with HIV (PLHIV) in the world; 25.6 million of the 38.4 million PLHIV are based in SSA. It is worth noting that despite notable decrease in new infections since the mid-2010 (new cases reduced by 32%), there were still approximately 1.5 million new infections in 2021, equating to an average of 4,000 new infections per day [2].

Globally, huge efforts have been made to prevent the spread of HIV, although the epidemic is still spreading. The only way to stop HIV transmission in the early stages of the epidemic was to use condoms. Treatment as prevention and test-and-treat [3] became crucial aspects of global HIV prevention strategies after effective ART was found to prevent HIV transmission to sexual partners [4] and led to the Fast Track strategy of UNAIDS to end the AIDS pandemic by 2030 [5]. Furthermore, UNAIDS established a worldwide strategy in 2011 to keep mothers with HIV alive and to end new HIV infections in children by 2015. This gives eliminating mother-to-child HIV transmission a top priority in the 22 countries where 90% of the world's HIV-positive pregnant women live [6]. Criteria to eliminate vertical transmission include a rate of 50 new child HIV infections or less per 100,000 live births and a HIV incidence of below 5% among breastfed infants [7]. According to a 2016 UNAIDS study, between 2009 and 2015, 1.2 million new HIV infections in children were prevented in the 21 SSA priority countries included in the global plan. Since 2009, the number of new HIV infections has decreased by more than 70% in seven countries (Swaziland, Burundi, Mozambique, Malawi, South Africa, and Namibia) [8].

The Middle East and North Africa (MENA) region was singled out for having inadequate data and an uncertain HIV/AIDS status [9]. However, HIV research in the MENA region has grown significantly in the last two decades, with some countries establishing surveillance systems to track the spread of HIV infection, particularly among those most at risk [10]. Egypt has a low HIV prevalence (< 0.01), however, there is evidence of a concentrated epidemic among two groups: Intravenous drug user (IDU) and men who have sex with men (MSM) [11]. Although the estimated number PLHIV in Egypt is modest, the incidence of HIV infections in Egypt is rising rapidly relative to other MENA countries, with a 25–30% annual increase in the number of new confirmed cases over the last 10 years [12, 13].

With available ART, HIV infection can be treated and the life expectancy PLHIV can be increased, as ART lowers HIV viral loads and delays the progression of the disease and consequently, increases the survival rate [14]. In 2020, UNAIDS estimated that around 10.2 million people worldwide were not on ART with 4.1 million of whom did not know their HIV-positive status and 6.1 million of those who knew their HIV status but could not access treatment [15]. In order to control HIV epidemic by 2020, UNAIDS set the 90–90-90 treatment goals in 2014. The 90–90–90 objectives stipulate that 90% of persons who live with HIV have been diagnosed as HIV positive, 90% of individuals who are HIV positive are receiving ART, and 90% of ART recipients have viral load suppression [16]. The results of modelling experiments demonstrated that if these goals are met, the number of new HIV infections could be reduced to levels that could lead to control of the HIV/AIDS epidemic by 2030 [15, 17].

In Egypt, the first ART became available in 2008. These were supported between 2008 and 2014 by the Global Fund to Fight AIDS, Tuberculosis, and Malaria (GFATM). The Ministry of Health and Population (MOHP) took the initiative in 2014 to begin covering ART from domestic funding, and it has been successful since 2017 in covering 100% of Egypt's needs. In 2018, the MOHP, took a significant step to ensure that PLHIV have access to necessary medication. They encouraged local pharmaceutical companies to register all first-line ART to cover approximately 97% of the drugs needed by PLHIV. This move by the MOHP is vital as it allows local pharmaceutical companies to produce and distribute first-line ART, making them more accessible and affordable for PLHIV [18, 19].

The HIV response in the MENA region is lagging behind global targets for prevention, testing, and treatment. This is due to various factors such as inadequate funding, insufficient monitoring, and prejudice. The situation is further complicated by the prevalence of conflict and humanitarian crises in the region, as well as the recent emergence of the coronavirus disease 2019 (COVID-19) pandemic [20, 21].

As the UNAIDS deadline approaches, HIV practitioners and programs are increasingly relying on the HIV care continuum to track progress and identify gaps, it is critical to assess country-level, regional, and global progress toward 90–90–90 targets on a regular basis. Identifying gaps can assist governments in making the required course adjustments to enhance and extend service delivery when necessary. There is scarcity in literature that highlighted the burden of HIV in Egypt and forecasted its statistics. Therefore, the objective of this study was to describe the HIV trend in terms of prevalence, number of PLHIV, elimination of vertical transmission, progress towards 90–90-90 in Egypt using UNAIDS data since 1990. Moreover, we forecasted these indicators from 2022 to 2024.

## Methods

### Study design

Time series analysis was performed on HIV indicators from Egypt records from 1990 to 2021. Data for this study were retrieved from the UNAIDS database [22], which provides information on HIV incidence, prevalence, testing, treatment, and prevention stratified by age and sex. Unfortunately, we did not find any data related to the third objective of the 90–90–90 anywhere including the UNAIDS dataset. Time-series is simply a set of data points indexed in a temporal order dedicated to creating statistical data. More specifically, they are used to anticipate the values of a series using the Autoregressive Integrated Moving Average (ARIMA) model [23].

### Operational definitions

According to the definitions of UNAIDS and World Health Organization (WHO) the following definitions of terms are adopted [24]:

- PLHIV who know their HIV status: Percentage of PLHIV who know their HIV status at the end of the reporting period
  $$=\frac{\text{Number of people living with HIV who know their HIV status}}{\text{Number of people living with HIV}}$$
- PLHIV on ART: Percentage and number of adults and children on ART among all adults and children living with HIV at the end of the reporting period. The count should not include people who have stopped treatment, died or emigrated to another country or who are otherwise lost to follow-up at the facility during this period.
  $$=\frac{\text{Number of people}\text{ on ART at the end of the reporting period}}{\mathrm{Estimated}\;\mathrm{number}\;\mathrm{of}\;\mathrm{people}\;\mathrm{living}\;\mathrm{with}\;\mathrm{HIV}}$$
- PLHIV who have suppressed viral loads: Number and percentage of people living with HIV who have suppressed viral loads at the end of the reporting period
  $$=\frac{\text{Number of people living with HIV in the reporting period with suppressed viral loads}\, \left(\leq1000/\mathrm{mL}\right)}{\mathrm{Estimated}\;\mathrm{number}\;\mathrm{of}\;\mathrm{people}\;\mathrm{living}\;\mathrm{with}\;\mathrm{HIV}}$$
- AIDS mortality: Total number of people who have died from AIDS-related causes per 100,000 population
  $$=\frac{\text{Number of people dying from AIDS-related causes}}{\text{Total population regardless of HIV status}}$$
- Mother-to-child transmission of HIV: Estimated percentage of children newly infected with HIV from mother-to-child transmission among women living with HIV delivering in the past 12 months = 
  $$=\frac{\text{Estimated number of children newly infected with HIV among children born in the previous 12 months to women living with HIV}}{\text{Estimated number of children delivered by women living with}\text{ HIV who delivered in the previous 12 months}}$$

### Data preparation and statistical analysis

The data was compiled with Excel and verified for completeness and consistency. Data was explored, cleaned, coded using R version 4.2 for Windows. The patterns of each selected indicator and their change were described numerically and graphically with line graphs plotted using points on the X-Y axis, where X is the time in year (from 1990 to 2021) and Y is the value of the selected indicator for each year in number or percent. The order of autoregression was represented by AR(p), the order of moving average by MA (q) and the degree of difference by I(d). We built ARIMA models using HIV case data reported in Egypt from 1990 to 2021 to predict the number of PLHIV, the percentage of women who will receive ART, number of HIV exposed children who will not be infected, and progress towards the 90-90-90 targets in the coming three years (2022-2024). We used the autocorrelation function (ACF) and partial autocorrelation function (PACF) graphs to calculate the degree of ARIMA and built an ideal model based on Akaike's information criterion (AIC) and Schwartz's Bayesian criterion (SBC). To validate the predictive model, we confirmed that the forecast errors were not correlated and that they were normally distributed with a mean of zero and constant variance [25].

## Results

### Prevalence and estimated absolute number of PLHIV in Egypt

**Fig. 1:**
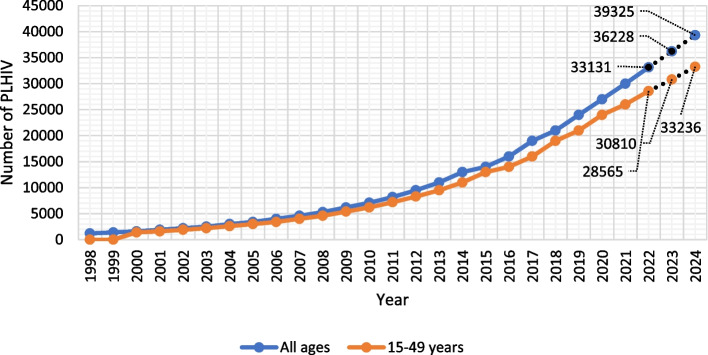
Observed number of people living with HIV in Egypt since 1998 till 2021 (blue line); number of adult people living with adults (15–49 years) HIV in Egypt since 1998 till 2021 (orange line). Dotted lines represent the predicted number of people living with HIV for 2022–2024

The prevalence of HIV among adults aged above 15 years including the following subcategories; adult males and females aged 15–49 years and young women and young men aged 15–24 years remain stable at a proportion of less than 0.1 since 1990. The total number of PLHIV (children and adults) was below 500 till 1993 then it increased to be < 1000 from 1994 to 1996. In 1996 this figure reached 1200 and gradually increased to be 30,000 in 2021. The number of children (0–14 years) living with HIV increased from < 100 to be < 200 in 1998. Then gradually increased to < 500 in 2003 and < 1000 in 2012. In 2021, the number of children living with HIV became 1100. The number of adult (15–49 years) living with HIV increased from < 500 in 1990 to be < 1000 in 1995. This number exceeded 1,000 to be 1200 in 1999 and increased gradually to be 26,000 in 2021 (Fig. 1). The number of PLHIV who are aged 50 years and older was < 100 for a decade (1990–2000), then increased to be < 200 from 2000–2005, reached < 500 from 2006–2010, increased to < 1000 from 2010–2014. In 2015 the number became 1100 and increased to reach 2900 in 2021. Based on these data the predicted number of PLHIV of all age groups will be 33,131 (95%CI, 32,316 – 33,947) in 2022, reaching 39,325 (95% CI, 33,236–37,334) in 2024. A similar predicted increase is found among adults aged 15 to 49 years; The number of cases is supposed to be 28,565 (95%CI, 27,607 – 29,522) in 2022, gradually increasing to be 33,236 (95%CI, 33,236 (95%CI, 30,553 – 35,920) in 2024.

### Elimination of HIV vertical transmission

**Fig. 2:**
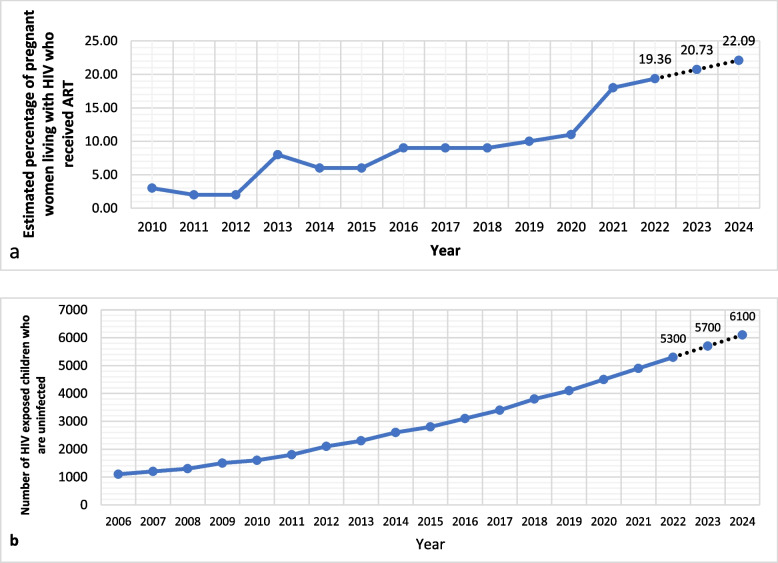
Elimination of vertical transmission: percentage of women who received ART. **a** number of HIV-exposed children who did not get infection. **b** Dotted lines represent the forecasted numbers of 2022–2024

The number of pregnant women who needed ART to prevent maternofetal HIV transmission increased from < 500 during 2010– 2014 to 780 in 2021. On the same line, the percentage of women who received ART increased from 3% (11 cases) in 2010 to 18% (141 cases) in 2021. We predicted that around 22% (95%CI, 13.0 – 32.0%) of pregnant females will have access to ART by 2024 (Fig. 2a). The number of children exposed to HIV who were not infected with HIV increased from < 100 in 1990–1991 to be < 200 in 1992 –1994. This number became < 500 from 1996 to 2000. In 2006 this figure exceeded 1000 and gradually increased to reach 4900 in 2021. Based on forecasting model, this figure supposed to be 5300 (95%CI, 5160 – 5439) in 2022 then increase steadily to be 6100 (95%CI, 5714 – 6485) in 2024 (Fig. 2b).

### Proportion of PLHIV who know their status [UNAIDS 90–90–90 first target]

**Fig. 3:**
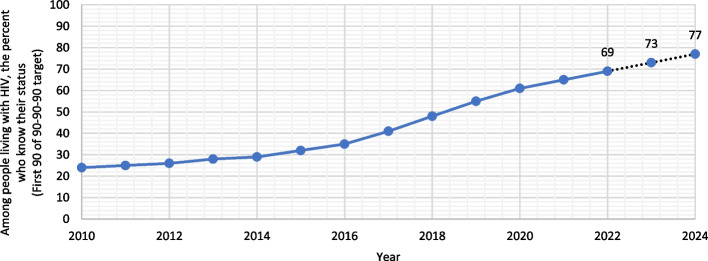
Among people living with HIV, the percent of the Egyptian patients who know their status (first target of 90–90–90). Dotted lines represent the forecasted numbers of 2022–2024

The percentage of population (all ages) who knew their HIV status increased from 24.0% in 2010 to 65.0% in 2021. The proportion of adults aged above 15 years who knew their HIV status has increased from 26.0% in 2010 to be 66.0% in 2015 (from 14.0% to 49.0% among females and from 36.0% to 73.0% among males). An increased number of children was observed during the same period from 7.0% in 2010 to be 26.0% in 2021. Using ARIMA model (1,2,0), the predicted number of patients who knew their HIV status in 2022 is expected to be 67.0% (95%CI 66.0–72.0), while the prediction of 2024 is 77.0 (95% CI 66.0–86.0) (Fig. 3).

### Proportion of PLHIV who know their status and on ART [UNAIDS 90–90–90 second target]

**Fig. 4:**
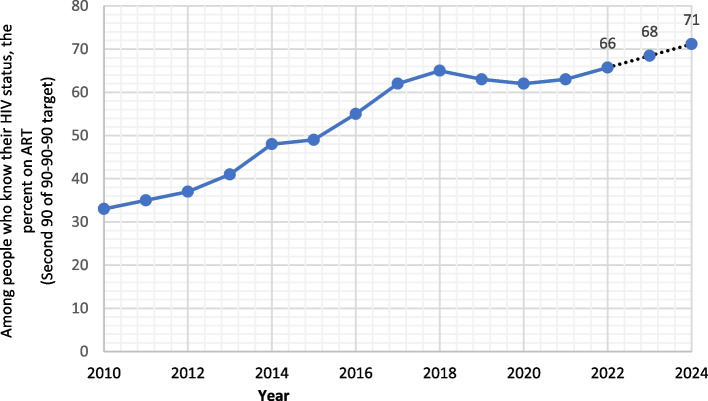
Among people living with HIV, the percent of the Egyptian patients who know their status and on ART (Second target of 90–90–90). Dotted lines represent the forecasted numbers of 2022–2024

Among all ages, the percentage of patients who knew their HIV status and were on ART increased from 33.0% to 63.0% between 2010 and 2020. The proportion of adults with HIV who knew their HIV status and were on ART increased from 32.0% in 2010 to 62.0% in 2021 with almost equal increase among males and females (32.0% to 63.0% vs 31.0% to 61.0%), respectively. Interestingly, this goal was achieved among children as more than 98.0% of children with HIV were on ART since 1990. However, in 2021 this number was suddenly decreased to 67.0%. Based on ARIMA forecasting model (0,2,0), the percentage of Egyptian patients with HIV who knew their status and on ART is supposed to increase to 66.0% (95%CI, 60.0%–72.0%) by 2022. Interestingly, this percentage is predicted to be 71.0% (95%CI, 61.0% – 81.0%) by 2024 (Fig. 4).

### AIDS related deaths

The overall mortality of AIDS per 1000 population is below 0.01 since 1990. However, AIDS-related deaths changed across different age groups. It was below 100 between 1990 and gradually increased to be < 200 in 2001–2005. The number of AIDS related deaths reached < 500 in 2006–2015, and finally the deaths increased to < 1000 between 2016 and 2021 except during 2018 and 2019 when the number of deaths decreased to < 500. Over the same time periods, the number of AIDS related deaths among children increased from < 100 in 1990 –2012 then increased to be < 200 since 2013 till now. Number of AID related deaths among adults aged 15–49 years remained < 100 from 1990–2004, then increased to be < 200 in 2005–2011, increased to be < 500 since 2012 until 2021. AIDs related deaths among people over 50 years remained stable since 1990 at a proportion of < 100.

### TB and HCV among HIV patients

Incidence of TB among patients with HIV was < 100 since 2000, while it increased in 2020 to be < 200. TB-related deaths among PLHIV increased from 15 to 45 between 2000 and 2020. All patients with HIV were tested for concomitant HCV infection in 2021.

## Discussion

In this study we used time series analysis to describe the HIV statistics in Egypt since 1990 and to forecast number of PLHIV, the percentage of women who will receive ART, number of HIV exposed children who will not be infected, and progress towards the 90–90–90 targets in the coming three years (2022–2024). This approach was used many times before to study the HIV epidemiology. A study by Guo et al., [26] used time series analysis to forecast the number of undiagnosed cases of HIV in China and assess the effectiveness of the implemented control measures. Similarly, Aboagye-Sarfo et al., [27] used the same approach to predict the incidence of HIV in Ghana within the coming 3 years. They found that the forecasted incidence exceeded the national HIV control program predicted incidence. Moreover, the ARIMA model was used to forecast the magnitude of opportunistic infection among HIV patients on ART in Uganda [28]. This highlights the importance of using such advanced statistical approaches to help policymakers and stake holder for better planning of national control program to combat HIV spread.

The number of PLHIV in Egypt remains relatively low (30,000 at the end of 2021) with a prevalence rate of < 0.01. However, the number of infected people is increasing rapidly: It increased five times since 2010 (from 5,400 to 30,000 cases). Based on the developed model, we expected that the number of PLHIV will be 39,000 by 2024. This mean the number of cases will increase by 30% within 3 years. This increase in the number of cases could be due to the multiple rounds of surveillance that have been conducted in Egypt on people who are at high risk of contracting HIV [10, 29]. In response to this increase in number of cases the MOHP implemented different preventive and control measures like including case-based surveillance in the health information system, development of HIV case surveillance system counting for reporting of ART initiation, and updating the national HIV monitoring and evaluation plan. Moreover, the MOHP has announced an increase in the number of treatment centers to twenty-seven centers nationwide which provide care and treatment services for HIV infected individuals. All services provided to persons living with HIV are free and confidential, including health awareness, and advice, medical examination, ART, and follow-up [30].

Egypt, a low middle-income country with a population of 100 million, had one of the highest hepatitis C virus (HCV) infections worldwide [31]. More than 2 million individuals were treated by 2018 (representing 40% of the entire HCV-infected population), with cure rates exceeding 90% [32]. When HCV infection occurs together with HIV, it significantly affects the course and outcomes of both illnesses [33]. Chronic HCV infection has the potential to increase the morbidity and mortality among PLHIV. HCV causes chronic inflammation, leading to impaired immune system recovery after ART. PLHIV are more likely to have a high viral load, and they face lower chances of spontaneous clearance of the HCV infection [34]. Furthermore, HIV worsens the effects of HCV and leads to accelerated progression to liver cirrhosis, liver cell failure, and hepatocellular carcinoma. The leading risk factor for the co-occurrence of HCV and HIV was the transfusion of blood and blood products. The prevalence of HCV and hepatitis B virus (HBV) infections is considerably higher in HIV-infected patients, with rates approximately 20 and 10 times higher, respectively, than in the general population without HIV infection. Recognizing these viral co-infections is crucial for developing appropriate treatment strategies [35]. Egypt is one of the few countries (Jordan, Egypt, Belarus, Azerbaijan, Georgia, Singapore, Seychelle, Montenegro, and Costa Rica) that has the highest HCV test rate among HIV patients. All patients with HIV are tested for concomitant infection with HCV infection [36]. This strategy may help to reduce the burden of both diseases and improve patient outcome and survival.

In Egypt, the number of AIDS related deaths is increasing; however, it is less than 1000 cases in 2021. On the contrary, the number of deaths among all ages in the MENA region decreased from 6600 to 5100 in 2021 [36].The cause of this upward trend in deaths may be due to the high dropout of treatment in the first year of the treatment protocol due to various personal and structural reasons, including lack of knowledge about treatment and fear of stigma [37, 38]. The age distribution among HIV infected Egyptians is changing over time. Children had a minor contribution to the number of PLHIV since 1990. However, from 2015–2020 there has been an observed increase in the number of infected children. This coincides with the increase in the number of children infected with HIV in the MENA region. In 2021 about 9100 children were tested positive for HIV compared to 8000 cases diagnosed in 2010 (11.3% increase in number of infected children in 11 years). The number of females living with HIV was slightly higher than males till 2009, when equal number of males and females were reported (2900), then the number of men living with HIV increased rapidly to reach 21,000 in 2021 while the number of females increased less steadily to be 7900 in the same year. The pattern over time in the MENA region was similar, except that the number of males living with HIV was higher throughout this period (1990–2021) and the pattern of increase in the number of males living with HIV was observed more in Egypt. This may urge the need of providing public health interventional program targeting males aged 15–49 to identify the risky behaviors that may increase their susceptibility to contract infection compared to females of the same age [36].

The Egyptian National AIDS Program (NAP) has implemented measures to support PLHIV during the COVID-19 pandemic. To ensure uninterrupted access to ART, the NAP has extended the dispensing interval beyond the usual one-month period. Additionally, teleconsultation services have been strengthened to provide remote medical support and follow-up for COVID-19-infected PLHIV. The NAP has also assisted in admitting infected PLHIV to isolation hospitals and providing subsequent care [18].

Egypt is moving towards achieving the 1^st^ and 2^nd^ targets of 90–90–90, however, this needs more effort to achieve the targets in time. According to the forecasting model, 77.0% of PLHIV will know their HIV status and 71.0% of those who know their HIV status will be on ART by 2024. These figures are not so far from that of the MENA, in 2020, 67.0% of the HIV population knew their status and 74.0% of them are on ART considering the progress of high-income countries in the region that have achieved most of the 90–90–90 goals. This delay in achieving the 90–90–90 targets in MENA including Egypt may be due to the overburdened health system and consequences of the COVID-19 pandemic that affected health system delivery all over the world [39]. Data from the MENA region revealed that 89.0% of HIV patients have achieved viral suppression. Unfortunately, no data was available about the third target in Egypt. Several studies have emphasized the obstacles of tracking progress toward 90–90–90, such as variations in reported statistics, limited data availability in the public domain, and lack of cross-country comparison of cascades [40, 41].

### Points of strengths and limitations

To the best of our knowledge this is the first study to describe different HIV indicators over long period in Egypt. However, the main limitation of this study is the unavailability of data like data of viral suppression, the third objective of the 90–90–90. In addition, some indicators were not expressed in absolute numbers (i.e. number of deaths < 100), this hindered modeling of some indicators like prevalence of HIV infection.

## Conclusion

In the MENA, HIV is growing very fast in term of incidence, new cases reported, prevalence, number PLHIV, and mortality. Similarly, the epidemiology of HIV is changing in Egypt since 1990. The number of PLHIV is increasing, especially among males compared to females. The number of pregnant women who need ART is increasing concurrently with an increase in the proportion of pregnant women receiving ART. This was reflected in the number of children who were exposed to HIV and were not infected. Egypt is implementing different preventive and control programs including the 90–90–90 strategy to control the diseases. There has been an observed increase in achieving the first and second targets of 90–909–90 in the past few years, and we predict more increase in the coming years. However, more efforts should be directed to achieve the target of the 90–90–90 in time. More stringent active and passive screening programs especially among population at risk may help to diagnose unidentified cases to limit spread of infection. Future research is mandatory to assess knowledge, attitude, and practices of Egyptians about HIV. Then based on these studies’ findings, delivering health messages through commonly used sources of information about the modes of transmission, magnitude of the problem, and importance to comply to ART is crucial.

## Data Availability

Data is available upon request by emailing the first author.

## Abbreviations

HCV: Hepatitis C virus
MOHP: The Ministry of Health and Population
ACF: Autocorrelation function
AIC: Akaike’s information criterion
AIDS: Acquired immunodeficiency syndrome
ARIMA: Autoregressive integrated moving average model
ART: Antiretroviral therapy
COVID-19: Coronavirus disease 2019
HIV: Human immunodeficiency virus
MENA: The Middle East and North Africa region
NAP: The National AIDS Program
PACG: Partial autocorrelation function
PLHIV: People living with human immunodeficiency virus
SBC: Schwartz's Bayesian criterion
UNAIDS: The Joint United Nations Program on HIV/AIDS
